# What influence do courses at medical school and personal experience have on interest in practicing family medicine? – Results of a student survey in Hessia

**DOI:** 10.3205/zma001217

**Published:** 2019-02-15

**Authors:** Antonia Bien, Gisela Ravens-Taeuber, Maria-Christina Stefanescu, Ferdinand M. Gerlach, Corina Güthlin

**Affiliations:** 1 Goethe University Frankfurt am Main, Institute of General Practice, Frankfurt/Main, Germany; 2 Frankfurt University Hospital, Clinic of Pediatric Surgery and Pediatric Urology, Frankfurt/Main, Germany

**Keywords:** family medicine, medical students, specialization in medicine, teaching, educational research

## Abstract

**Aim: **Against the background of an impending shortage of family practitioners, it is important to investigate the factors influencing the choice to become one. The aim of this study was to identify factors that encourage medical students to choose to practice family medicine.

**Method:** Using a questionnaire, students in the fourth and fifth years of their studies in the Federal State of Hesse were asked about the factors that had influenced their choice of medical specialty and their experience of courses in family medicine. Predictors of an interest in practicing family medicine were calculated using multiple logistic regression.

**Results: **361 questionnaires were returned, representing a response rate of 70.9%. Confirmation of personal strengths, an interest in the field, and practical experience of the subject generally turned out to be important factors influencing the choice of medical specialty. 49.3% of students expressed an interest in practicing family medicine. A link existed between an interest in working as a family doctor and the opportunity to take over an existing practice, experience of medicine in rural areas, and an appreciation of the conditions of work. With regard to education at medical school, positive experiences during a clinical traineeship in family medicine and positive role models among teachers of general practice were identified as predictors.

**Conclusion: **Almost half the medical students were open to the idea of practicing family medicine. Experience of medicine in rural areas and positive experiences of courses in general practice were linked to an increased interest in working as a family doctor. To promote this interest, it may be a promising approach to increase opportunities to collect experience of medicine in rural areas, and to encourage highly motivated teaching practices.

## Zusammenfassung

**Zielsetzung:** Vor dem Hintergrund des drohenden Hausärztemangels kommt der Untersuchung von Einflussfaktoren auf die Wahl einer hausärztlichen Tätigkeit große Bedeutung zu. Ziel der Studie war es, Faktoren bei Medizinstudierenden zu detektieren, die die Wahl einer hausärztlichen Tätigkeit begünstigen.

**Methodik: **Mittels Fragebogen wurden Studierende im 4./5. Studienjahr im Bundesland Hessen zu Einflussfaktoren auf die Wahl der Weiterbildungsrichtung und Erfahrungen mit allgemeinmedizinischen Lehrveranstaltungen befragt. Prädiktoren für ein Interesse an einer hausärztlichen Tätigkeit wurden mittels multipler logistischer Regression berechnet.

**Ergebnisse: **Mit 361 ausgefüllten Fragebögen wurde eine Rücklaufquote von 70,9 % erreicht. Die Bestätigung eigener Stärken, das Interesse am Fachgebiet sowie praktische Erfahrungen im Fach stellten sich als wichtige Einflussfaktoren auf die Wahl der Weiterbildungsrichtung generell heraus. 49,3% der Studierenden äußerten Interesse an einer hausärztlichen Tätigkeit. Es zeigte sich ein Zusammenhang zwischen dem Interesse an einer hausärztlichen Tätigkeit und einer in Aussicht stehenden Praxisübernahme, Erfahrungen mit Medizin in ländlichen Regionen und einer Wertschätzung der Arbeitsbedingungen. Im Bereich der allgemeinmedizinischen Lehre konnten positive Erfahrungen mit einer allgemeinmedizinischen Famulatur sowie positive allgemeinmedizinische Rollenvorbilder als Prädiktoren identifiziert werden.

**Schlussfolgerung: **Fast die Hälfte der Studierenden ist einer hausärztlichen Tätigkeit gegenüber aufgeschlossen. Vor allem Erfahrungen mit Medizin in ländlichen Regionen sowie positive Lehrerfahrungen in der Allgemeinmedizin zeigten einen Zusammenhang mit einem gesteigerten Interesse an einer hausärztlichen Tätigkeit. Um dieses Interesse weiter zu fördern, könnten somit ein Ausbau von Erfahrungen mit Medizin in ländlichen Regionen sowie eine Förderung engagierter Lehrpraxen erfolgversprechend sein.

## 1. Introduction

### 1.1. Background

In the German healthcare system, there is an increasing shortage of doctors in certain specialties and regions [1], [2]. In the outpatient sector, family health care has been the most seriously affected in this respect. While the proportion of medical specialists rose from 39.9% to 54.1% between 1991 and 2012, the proportion of family practitioners sank from 60.1% to 45.9% [3].

Unless appropriate countermeasures are taken, demographic change will lead to further deterioration in the situation, as a growing prevalence of chronic diseases among the elderly is expected to increase the demand for family medical care [3].

An increase in demand is occurring at the same time as an interest in setting up in private practice is declining, and this is particularly true for medical specialists in general practice. [3], [4]. A significant rise in the average age of practicing family doctors and the desire of many young doctors to work part time are further aggravating the shortage [4]. By 2025, up to 20,000 additional family practitioners will be needed to ensure the continued provision of comprehensive health care in family medicine [3].

#### 1.2. Problem 

To satisfy the need for family practitioners in Germany, between a quarter and a third of all graduates from German medical faculties would have to seek to specialize in this field. 20% to 76% of students nationwide [5], [6], [7], [8] declare an interest in general practice but slightly below a tenth of students consider family medicine to be their clearly preferred medical specialty [7], [8], [9]. A longitudinal study conducted at Hamburg University revealed that only around 10% of graduates actually begin to specialize in family medicine [10]. Against this background, it is very important to conduct an investigation into the necessary conditions and the influencing factors that would encourage future doctors to choose to specialize in family medicine. 

#### 1.3. State of research

International research has paid considerable attention to the multifactorial influences on the choice of family medicine as a medical specialty. In Germany, on the other hand, very few studies exist and most of those that do focus on individual aspects. The factors that have been identified in international literature as being linked to the choice to specialize in family medicine can be broken down into six categories [11]. These are presented in table 1 (Tab. 1).

#### 1.4. Aim

The aim of the present study was to identify and detail the factors that influence the choice to work as a family doctor among medical students in the late stages of their studies in the German state of Hesse, and to compare them with one another in terms of importance. A special focus was placed on influences from the field of personal experience, as well as experiences with courses in general practice, and with teachers. 

## 2. Methods

### 2.1. General conditions and sample

Based on the results of a comprehensive literature search and our own ideas, a questionnaire (see attachment 1 ) was developed that contains questions on known and published factors that influence interest in becoming a family practitioner, as well as parameters that have not yet been investigated. The questionnaire was handed out to students of medicine at medical faculties in the state of Hesse (Frankfurt, Marburg, Gießen) between July 2015 and April 2016. In Frankfurt and Marburg, it was distributed immediately following the two-week curricular internship in a family practice, and generally in person at the final meeting. In Gießen it was distributed after a final exam, several weeks after the internship had taken place. 

The students were in the fourth or fifth year of their six-year education. 

#### 2.2. Measuring instrument

The questionnaire can be divided into four sections.

The first section deals with how the students see their future professional lives. The second asks about their experiences with courses in general practice and the teachers themselves. This section was adapted to take account of the courses at each individual university. The third section deals with the image that family medicine has among medical students. Section 4 asks about demographic factors, such as age, gender, and family status. Overall, there are 26 questions, of which 2 are free-text, 10 are single choice, 6 multiple choice and 8 questions require answers on a 5-point Likert scale (1=don’t agree at all/doesn’t apply at all to 5=completely agree/fully applies)

The questionnaire was pilot-tested based on expert opinions and five cognitive interviews, and then revised as appropriate. 

#### 2.3. Data analysis

The data were evaluated using SPSS 22.0 (IBM). In addition to a descriptive analysis, the link between interest in specializing in general practice and selected items was analyzed. Previously operationalized interest, expressed by having named family medicine as the first or second choice of medical specialty, or a Likert scale rating of "4" or "5" to the question whether practicing family medicine was conceivable, was used as a dependent variable in the stepwise multivariate logistic regression that was then carried out. Independent variables were selected when they showed a significant correlation with interest in general practice in bivariate models. Ten out of the 21 items that were subjected to a bivariate investigation were included in the multivariate logistic regression (see table 2 (Tab. 2)). As the item "age" had revealed itself to be clearly non-significant in the bivariate model, it was not taken into account in the multivariate model. The factors gender, size of hometown, family status and previous professional training were included in the model as control variables. For the regression model, p=0.05 was considered to indicate statistical significance. 

## 3. Results

### 3.1. Sample description

**Figure 1:**
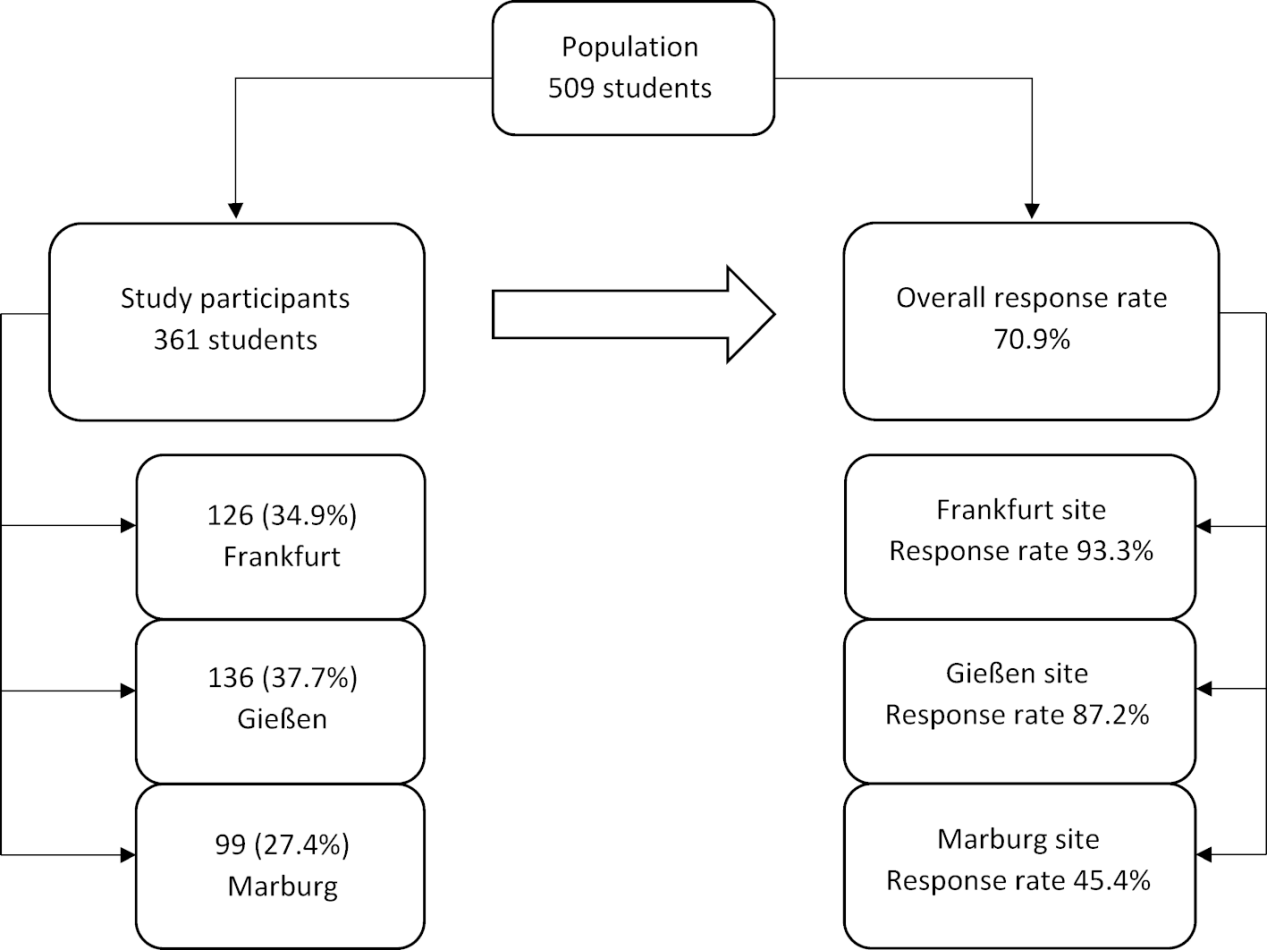
Distribution of study participants among university sites with corresponding response rates.

A total of 361 questionnaires could be included in the analysis, representing a response rate of 70.9%. The response rate varied considerably, depending on faculty location (see figure 1 (Fig. 1)). Participants’ sociodemographic characteristics are presented in table 3 (Tab. 3).

#### 3.2. Ideas on future professional career

**Figure 2:**
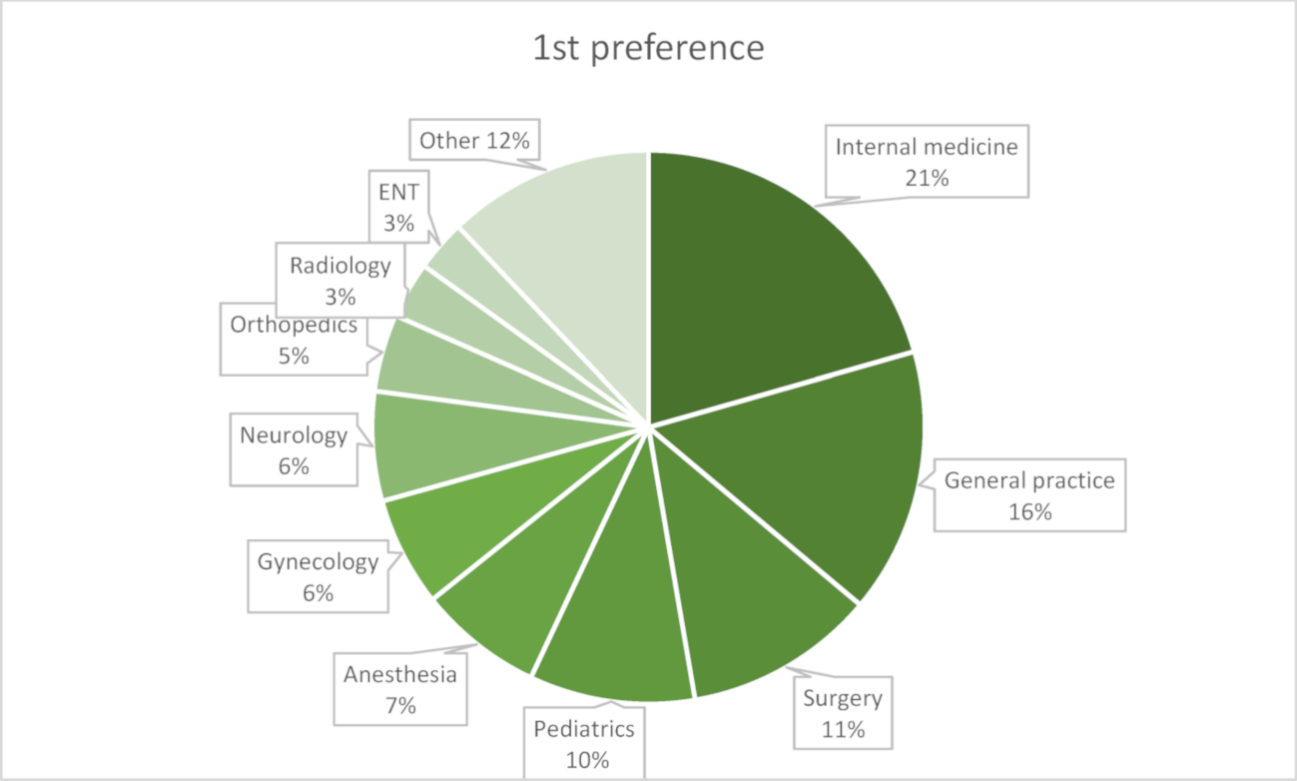
Distribution of medical specialty preferences among students: first preference

**Figure 3:**
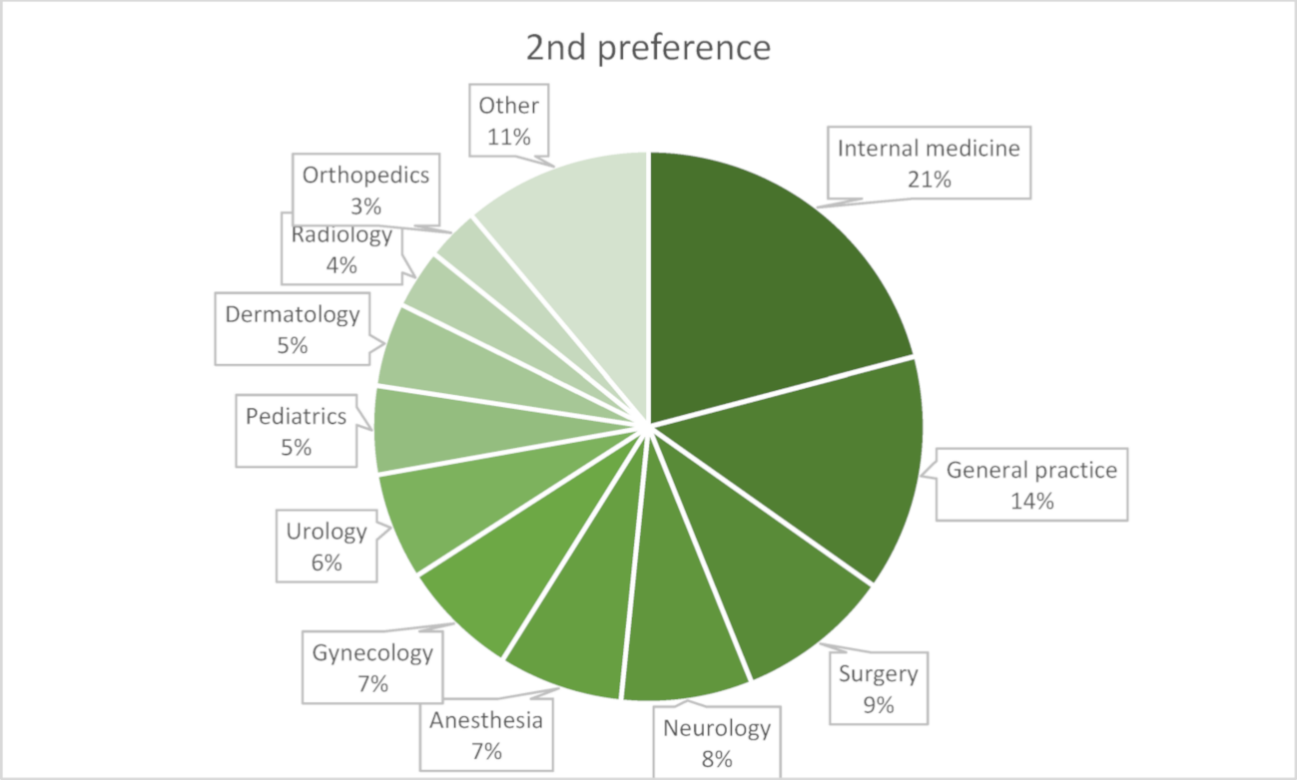
Distribution of medical specialty preferences among students: second preference

The question about preferred medical specialty is key to assessing interest in practicing family medicine (see attachment 1 , question 1). Up to two alternatives could be mentioned. 91.9% of students had specific ideas which specialty(ies) they preferred, and only five could imagine working outside patient healthcare, for example in the pharmaceutical industry. The three most popular medical specialties were internal medicine, general practice, and surgery (see figures 2 (Fig. 2) and figure 3 (Fig. 3)). The majority of students (60%) said their preferences had developed during their studies. 

#### 3.3. Influencing factors that were considered relevant to the choice of preferred career

The factors that were most relevant in the choice of preferred career were interest in the subject (mean (M) 4.63, standard deviation (SD) 0.68, response options 1 to 5 on a Likert scale), confirmation of personal strengths or skills (M 4.22 SD 0.81) and practical experience in the field (M 4.13 SD 0.93).

#### 3.4. Interest in family medicine

Overall, 49.3% of students said they were interested in practicing family medicine. Around a quarter of students specifically mentioned general practice as their first or second choice of medical specialty. Almost half the students said they could imagine practicing family medicine at a later date, either as a specialist in general practice or in internal medicine (see attachment 1 , question 3).

#### 3.5. Experiences with teachers

More than half the students said their attitude towards the subject had been positively influenced either by general practice teachers, or by role models among teaching personnel (see table 4 (Tab. 4)). However, 39.1% of students said they found the attitude to general practice of other medical specialists to be rather disparaging. 

The students‘ evaluation of individual courses in general practice was mostly positive, especially of the internship (Mean 3.88, SD 1.13) and clinical traineeship (Mean 3.8 SD 1.12).

90.1% of students said their general practice education had lacked certain information, particularly with respect to the business aspects of running a private practice, ideas on how to manage/organize a practice, ways of finding one’s own practice, and different working time models. 80.1% of students thought this additional content should be provided in the form of voluntary courses. 

Almost half the students thought courses in general practice were a valuable part of their medical education. 62.5% said the insight provided into family medicine was sufficient for them to decide whether to consider or to rule out specializing in the field. 

#### 3.6. Comparison of students in terms of interest in family practice

A comparison between those students that showed an interest in practicing family medicine and those that did not revealed significant differences. An interest in working as a family practitioner was more common among those that said that working conditions in the field, experience of working in medicine in rural areas, and the opportunity to take over a practice had played an important role in the development of their preferences. 

As far as medical education is concerned, two further factors were significant. Both the view that doctors working in family medicine had a positive influence on personal attitudes towards family practice, and a positive evaluation of a clinical traineeship in general practice correlated positively with interest in working as a family practitioner (see table 2 (Tab. 2)). 

## 4. Discussion

### 4.1. Interest in practicing family medicine

49.3% of medical students expressed an interest in working as a family practitioner. Among preferred medical specialties, family medicine was the second most popular, after internal medicine. The results of our survey are comparable with the results of other German studies. In a survey by Deutsch et al., family medicine also took second place among preferred specialties [12]. In Kopp et al., 37.3% of participants showed an interest in practicing family medicine [8], while in Böhme et al., as many as 68.2% expressed such an interest [13]. It is worthy of note that the percentage of those interested in practicing family medicine varied considerably between studies. Interest declined in line with the specificity of the question on career aspirations, with scarcely a tenth of students describing family medicine as their clearly preferred medical specialty [9].

#### 4.2. Influencing factors on choice of medical specialty and interest in practicing family medicine 

The present study was able to show that experience of medicine in rural areas had a significant influence on interest in practicing family medicine, confirming data from Deutsch et al. [12]. Confirmation of personal strengths was also an important influencing factor in the development of future career aspirations in general. This new aspect should be investigated in more detail in future studies. A clear link between interest in practicing family medicine and the opportunity to take over a practice in the future has not previously been mentioned in the literature either.

Furthermore, students that expressed an interest in practicing family medicine were far more likely to say that their choice of specialty was influenced by working conditions. These data confirm the results of numerous international studies in which a link exists between interest in family medicine as a medical specialty and the appreciation of working conditions that permit a weaker career orientation, a marked focus on work-life balance [14], [15], [16], the reconciliation of work and family life [8], [17] and regular working hours [8], [18]. 

As in Ko et al., personal interest and practical experience in field had a significant influence on choice of medical specialty in general [19]. 

#### 4.3. General practice courses and interest in practicing family medicine 

With regard to courses in general practice, our study showed a link between both positive experiences during the clinical traineeship in general practice, and positive role models among family practitioners, and an increased interest in practicing family medicine. The positive influence of certain courses and especially the internship in general practice has already been described in numerous surveys [13], [20], [21], [22]. Whereas the influence of experiences gathered during clinical traineeships in general practice has only been described in one other study [23].

However, the quality of courses depends on the teachers themselves. Good supervision by the teaching physician is linked to an increasing likelihood that students will choose to specialize in family medicine [24]. Positive role models generally have a significant influence on the choice of medical specialty [25], [26]. Qualitative studies have often described positive role models as being the most important influencing factor [27], [28], [29], but up to now, this factor has only been surveyed quantitatively in two German studies [12], [17]. More than half the participants either said they had found a positive role model among the teaching physicians in general practice, or that family practitioners had had a positive influence on their attitude towards family medicine. Agreement with this statement also correlates significantly with interest in practicing family medicine. We can thus conclude that doctors practicing family medicine have a positive influence on the choice of the subject as a medical specialty.

#### 4.4. Demographic aspects and interest in practicing family medicine

The link between demographic aspects such as gender and age and an interest in specializing in family medicine that has been described in comparable studies [6], [8], [14], [30] could not be confirmed in our study. It is possible that no link with age could be identified because there was little variance in the age of study participants.

#### 4.5. Possible reasons not to choose to practice family medicine 

Natanzon et al. described the negative influence of "badmouthing" in their study [31], and indeed, 39.1% of participants in ours said they had found other medical specialists to have a rather negative attitude towards family medicine. However, as only 14.8% of students said their attitude towards family medicine had been negatively influenced by specialists in other fields of medicine, this effect does not appear to have played an important role in our sample. This was confirmed in two further studies [28], [32]. Apart from the "badmouthing" aspect, Natanzon et al. also mentioned that little contact with family medicine was a further reason to decide against specializing in the field [31]. In a further study, doctors training to specialize in family medicine said family medicine should play a more substantial role in medical studies [16]. Despite high levels of satisfaction with existing courses in general practice, our results showed that some general practice content was not dealt with in sufficient detail. In this respect, the business aspects of setting up in private practice, different ways of running and organizing a practice, setting up a private practice, and flexible working hour arrangements, are particularly worthy of mention. Participants in a study by Buddeberg-Fischer et al. also said they lacked knowledge about business administration [33]. According to the students, additional course content should be provided as an option. Furthermore, 65.2% of students said their knowledge of family medicine was sufficient either to rule out, or to consider, specializing in family medicine in the future. 

#### 4.6. Strengths/weaknesses 

In our study, we surveyed students immediately following an internship in general practice. It is therefore possible that data collected at a different time point would have revealed a lower level of interest in family medicine. 

The personal distribution of a paper-based questionnaire was limited to the state of Hesse in order to achieve a higher response rate. 

It was not always possible to distribute the questionnaires in person, although the response rate in situations in which it was, was significantly higher. This led to considerable discrepancies in response rates, to the disadvantage of one site in particular. 

The aim of the study and the participation of the Institute of General Practice was mentioned before the questionnaires were distributed. It is therefore possible that although the data was anonymized, social desirability may have played a role. Furthermore, even if it was piloted in cognitive interviews, the questionnaire was still self-developed.

The item "experience of medicine in rural areas" was not explained in more detail in the questionnaire and there was no mention of where experiences were gained. An investigation into this aspect could have provided interesting insights. However, we were interested in general contact with medicine in rural areas, independently of experiences gained during studies and the students’ backgrounds. 

Overall, the response rate was extraordinarily high for a voluntary survey. The study dealt with many factors that could conceivably influence the choice of medical specialty. This enabled us to investigate quantitative aspects of the profiles of students that were interested in practicing family medicine in considerable detail. 

## 5. Conclusions

The current shortage of young general practitioners and a growing interest in working part time [34] mean it is necessary to stimulate an interest in family medicine among students of medicine. Many projects and measures have already been initiated, such as specialist training networks and competence centers in general practice. However, this does not appear to be enough, as barely 10% of students are currently certain that they will choose to practice family medicine [8], [35]. However, numerous other surveys have shown that general interest in the subject is significantly higher. This indicates that despite substantial interest in family medicine overall, certain contributing factors appear to influence the decision for or against family medicine during the course of medical studies, or immediately afterwards. Our results have shown a marked correlation between experiences with medicine in rural areas and an interest in practicing family medicine. It is therefore important to offer more opportunities for students to gather experience working in rural medical practices. Several pilot projects exist such as the "rural outing 2.0" that has been launched by Goethe University Frankfurt, which provides students with the opportunity to experience medicine in rural areas first hand, while at the same time dispelling any monetary and organizational concerns in advance. Similar projects should be established throughout the country, and existing projects should be developed further. This study was also able to demonstrate that personal experience and impressions gathered while attending courses in general practice are directly linked to an interest in practicing family medicine. Positive experiences with the subject should therefore be encouraged and, as positive learning experiences and good teachers can raise interest in practicing family medicine, a special focus placed on recruiting and supporting highly motivated and rated teaching practices [36]. One way of stimulating interest in general practice and in practicing family medicine may be the implementation of interest groups among students [37]. These could be used to provide information that is not taught during medical studies, promote contact with highly motivated teaching doctors and to improve the image of family medicine among students and of other medical specialists. 

## Acknowledgements

I would like to thank Prof. Dr. Erika Baum (Marburg University), Dr. Thomas Karg and his secretary Margarete Schulze (Gießen University) for their great help in collecting data. I would also like to express my gratitude to Linda Barthen, Dr. Nadja Becker, Dr. Marischa Broermann, Dr. Benita Mangold and Dr. Monika Sennekamp for their support in reviewing the questionnaire, and to Phillip Elliott for translating the manuscript into English. 

## Data

Data for this article are available from the Dryad Digital Repository: https://datadryad.org//review?doi=doi:10.5061/dryad.74tk6cr [38]

## Competing interests

The authors declare that they have no competing interests. 

## Supplementary Material

Questionnaire

## Figures and Tables

**Table 1:**
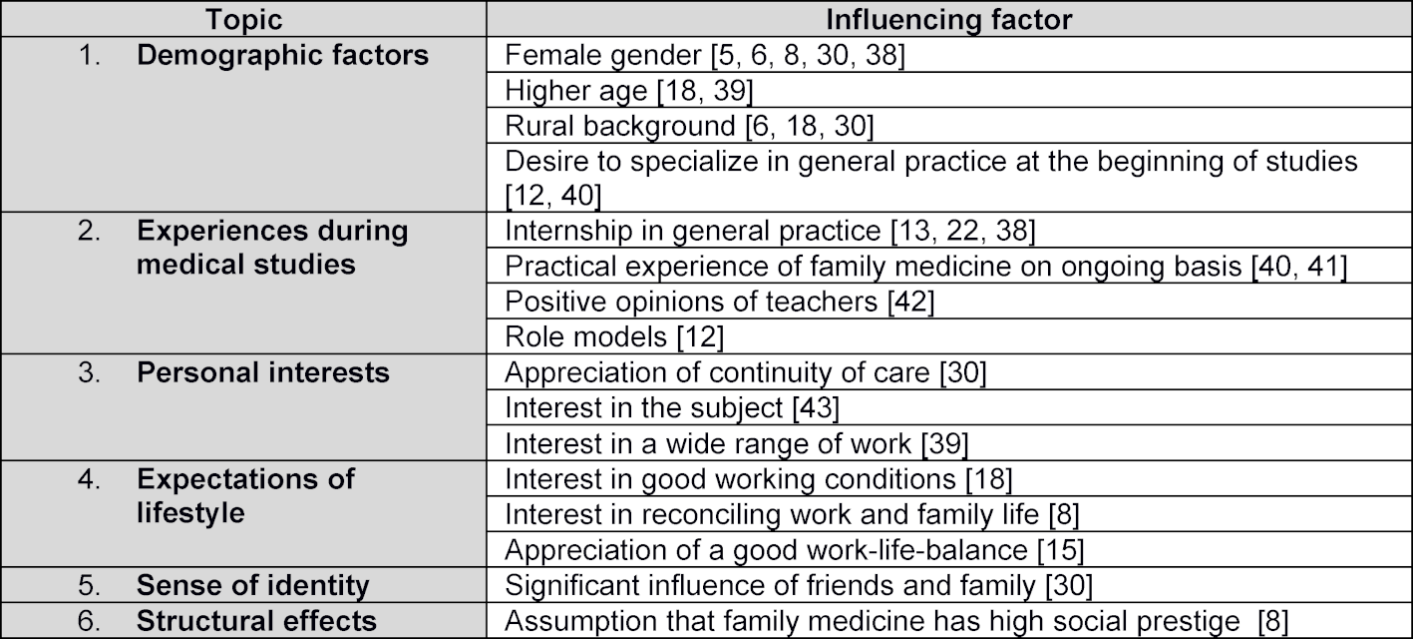
The main factors known to increase interest in practicing family medicine

**Table 2:**
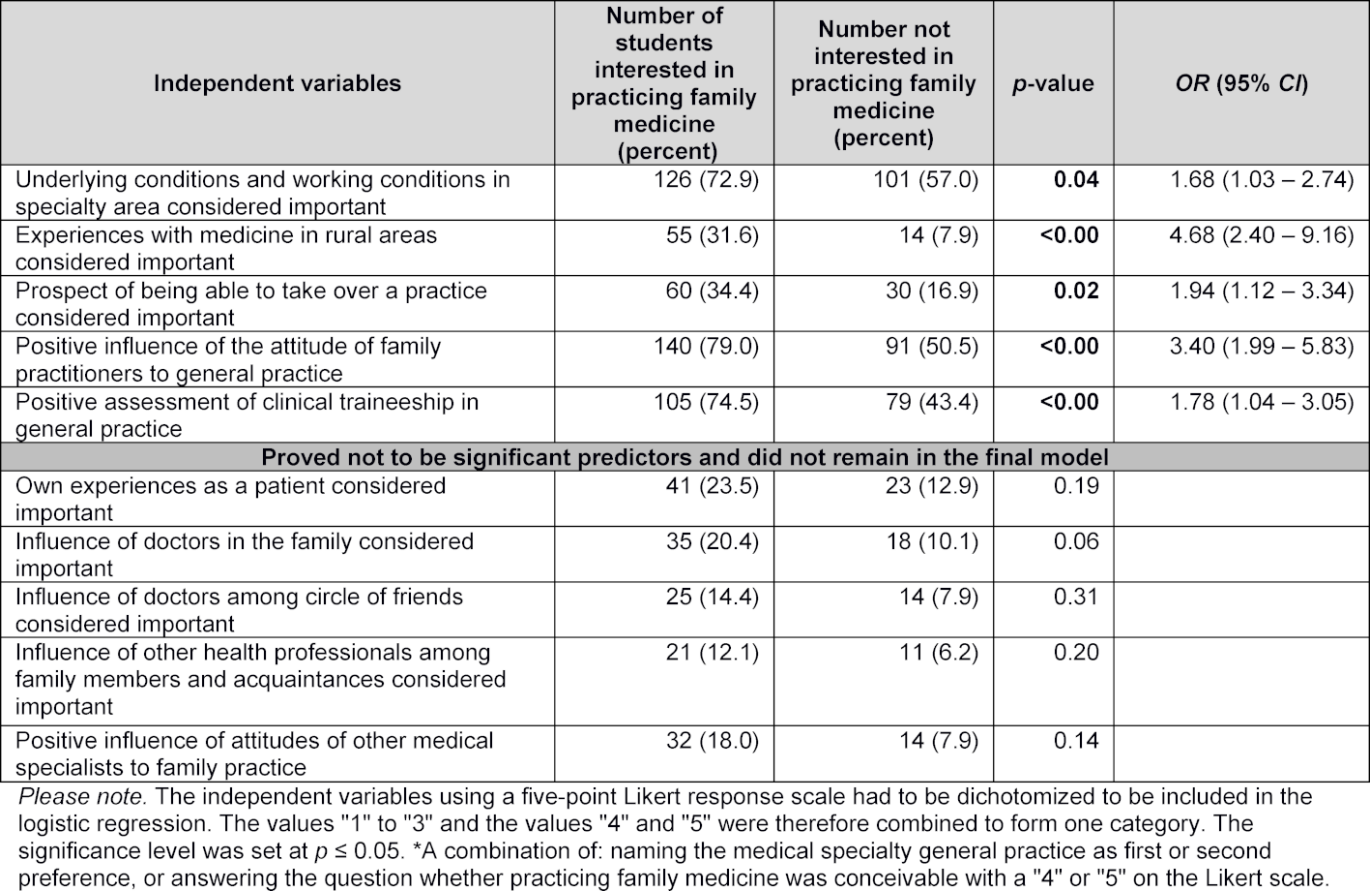
Results of multivariate logistic regression using the forward-stepwise method with the dependent variable "interest in practicing family medicine"*

**Table 3:**
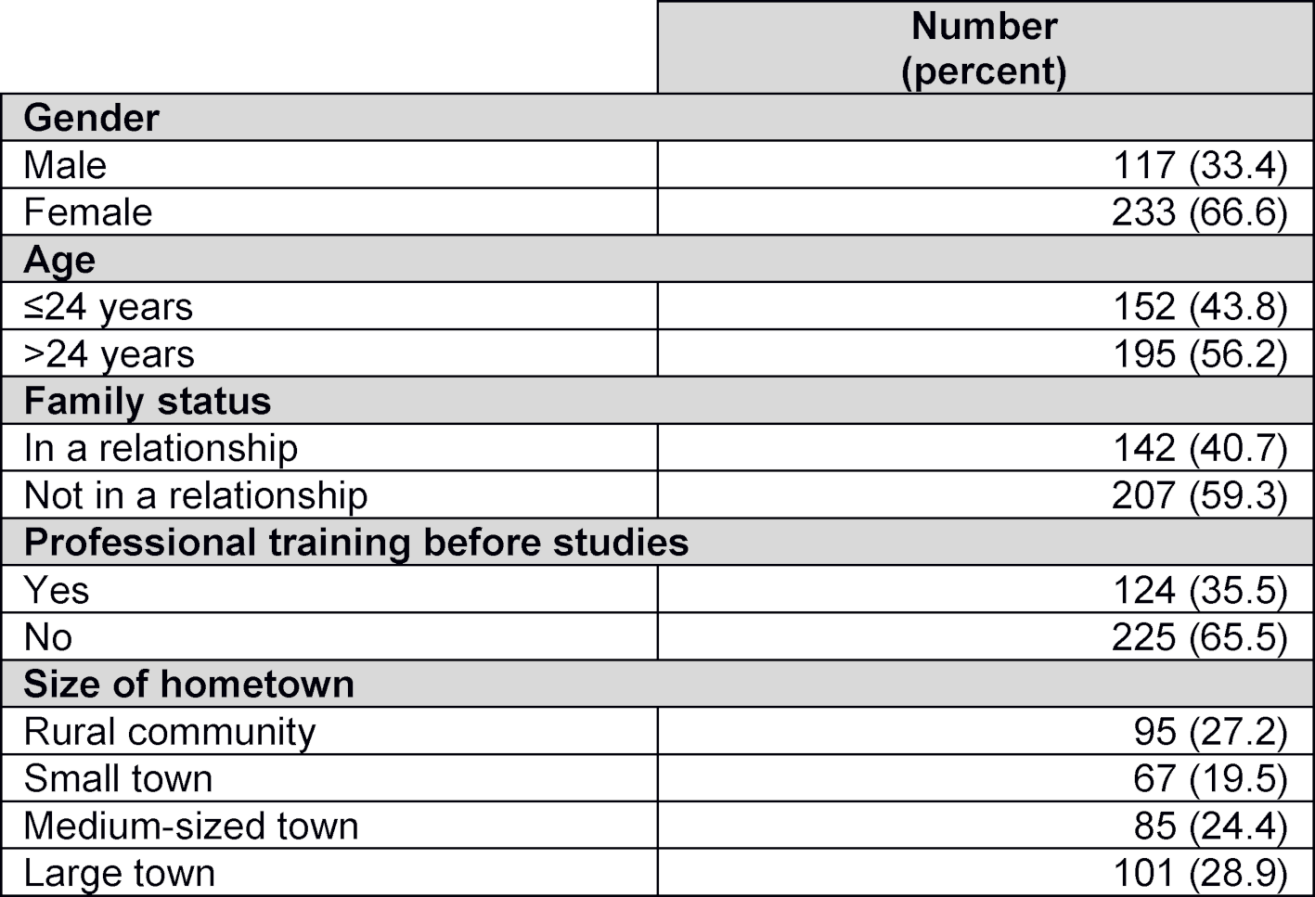
Sociodemographic characteristics of study participants

**Table 4:**
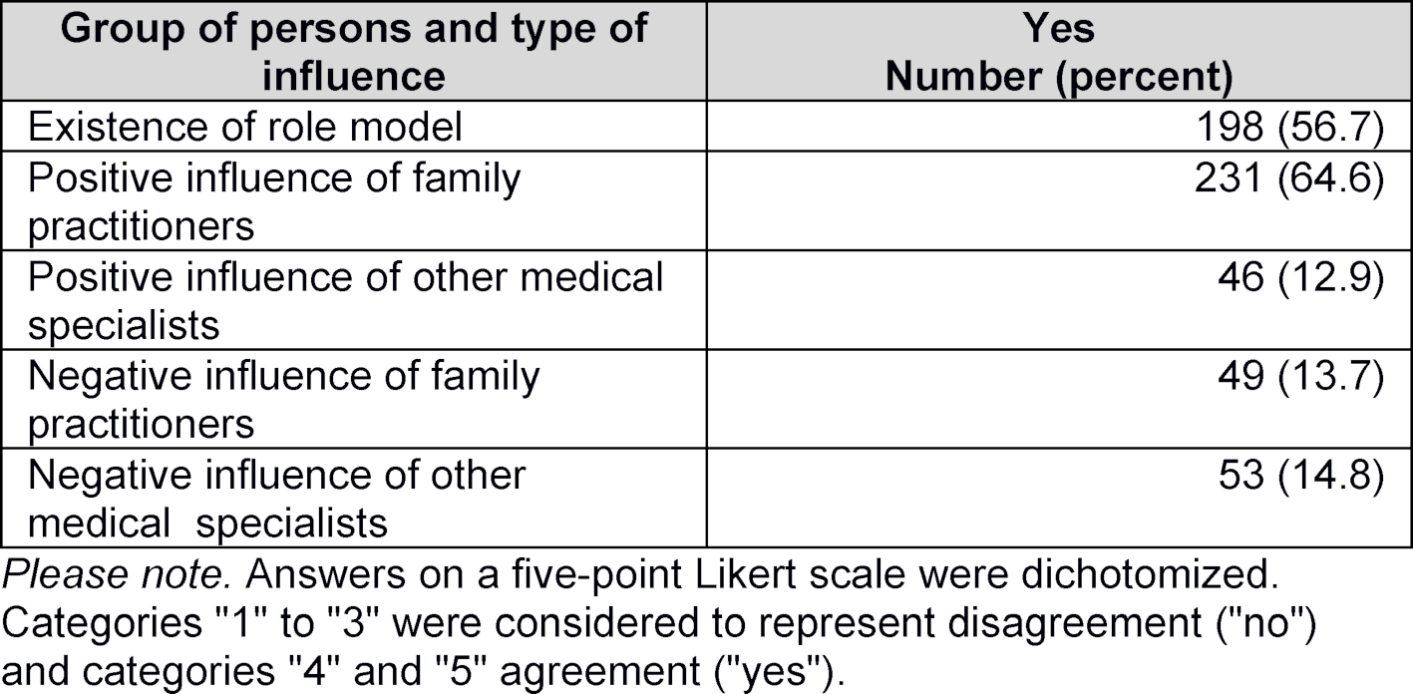
Experience of teachers of general practice
