# Supplementary material for: What influence do courses at medical school and personal experience have on interest in practicing family medicine? – Results of a student survey in Hessia
Source: GMS J Med Educ. 2019 Feb 15;36(1):Doc9. doi: 10.3205/zma001217 (PMC6390086; doi:10.3205/zma001217)
Supplement: Questionnaire [file JME-36-9-s-001.pdf]

Dear fellow students,

following questionnaire is part of my dissertation at the institute for family medicine in Frankfurt, which I ask you to complete.

The questionnaire is about your image of family medicine, about the aspects you like or dislike about family medicine and about the factors playing a role in the decision for or against family medicine as further medical specialist education.

Regarding the shortage of family physicians and doctors in rural areas it is the aim of this study to shape the education and the working conditions in a more attractive way. So it is another aim, to create a general basis for improvements of your own education and working conditions. You can participate in giving your answers and opinions and perhaps become clearer about your expectations of working life.

Irrespective of whether or not you like family medicine, all opinions are important for this study!

Completing the questionnaire will last 10 to 15 minutes and of course your data will be treated confidentially and pseudonymized evaluated.

If you have any questions or remarks please contact me under following mail-address: Antonia.Bien@gmx.net

Thank you very much for your support!

Antonia Bien

Contact:
Doktorandin cand. med. Antonia Bien
Institut für Allgemeinmedizin
Johann Wolfgang Goethe-Universität
Haus 10 c
Theodor-Stern-Kai 7
D-60590 Frankfurt
E-Mail: Antonia.Bien@gmx.net
Telefon:
Fr. Dr. C. Güthlin
Tel.: 069-6301-83882

This part is about your future working ideas

1. **Which medical specializations are you interested in in the order of your preferences?**
   (2 alternatives possible)

   1. ________________________________
   2. ________________________________

   ❐ I do not know yet

   ❐ None, but:

   ____
   (for example working in the pharma-industry)

2. **When did your medical specialization preference emerge?**
   (Multiple choice possible)

   ❐ Was certain previous to studies
   ❐ Was reinforced during studies
   ❐ Emerged during studies
   ❐ Emerged in an internship during studies

3. **Are you ready to set up in private practice / be employed as a family doctor (both specializing in family medicine or specializing in internal medicine but working as GP)**

**I can't imagine that at all**	☐ 1	☐ 2	☐ 3	☐ 4	☐ 5	**I can well imagine that**

4. **How important were the following factors or persons in the emergence of the desired field of work?**

	**Not important at all**				**Very important**
	**1**	**2**	**3**	**4**	**5**
Interest in the subject	☐	☐	☐	☐	☐
Confirmation of personal strengths and capabilities	☐	☐	☐	☐	☐
General / Working conditions in the preferred specialist area	☐	☐	☐	☐	☐
Negative/ disappointing experiences in other specialist areas	☐	☐	☐	☐	☐
Prospect of taking over or working in an existing practice	☐	☐	☐	☐	☐
Teaching in the preferred specialist area	☐	☐	☐	☐	☐
Practical experience in the specialist area	☐	☐	☐	☐	☐

	Not important at all				Very important
	1	2	3	4	5
Sense of achievement when gaining practical experience	☐	☐	☐	☐	☐
Taking on responsibility during practical training /clinical traineeships	☐	☐	☐	☐	☐
Experience of medicine in rural areas (inpatient and outpatient sectors)	☐	☐	☐	☐	☐
Personal experiences as a patient	☐	☐	☐	☐	☐
Influence of doctors in the family	☐	☐	☐	☐	☐
Influence of doctors among circle of acquaintances	☐	☐	☐	☐	☐
Influence of family members or acquaintances in another health-related profession (nurse, physiotherapist, etc.)	☐	☐	☐	☐	☐
Influence of family and acquaintances with no medical background	☐	☐	☐	☐	☐
Other: ________________________________	☐	☐	☐	☐	☐

5. In your opinion, what is to be said against… (Multiple choice possible)

**working in a practice?**	**a permanent occupation in hospital?**
❒ Financial risk	❒ Working in shifts
❒ Bureaucracy, administrative effort	❒ Competition
❒ Inadequate payment	❒ Hierarchy
❒ Recourse claims	❒ Lack of lasting patient contacts
❒ High workload	❒ High workload/ stress
❒ Difficulties getting a licence in preferred region	❒ No flexibility
❒ High responsibility	❒ Limited individual responsibility
❒ No career chances	
❒ Lack of contact with colleagues	
❒ Other: ________________________________	❒ Other: ________________________________

6. **a) Are you ready to work in a rural area in future?**

- ❐ Yes; go to **6b**
- ❐ No; go to **6b**
- ❐ I do not know; go to **7**

b) When did your attitude toward working in a rural area emerge?
(Multiple choice possible)

- ❐ Was certain previous to studies
- ❐ Was reinforced during studies
- ❐ Emerged during studies
- ❐ Emerged in an internship during studies

7. **From your point of view, pros and cons:**

**For working in a rural area as a family doctor**	**Against working in a rural area as a family doctor**
❐ More individual responsibility	❐ High workload
❐ Varied tasks	❐ Lack of contact with colleagues
❐ Close patient contact	❐ No privacy
❐ Safe job	❐ Lack of private patients
❐ Family friendly environment	❐ Difficulties for the partner in finding a job
❐ low living expenses	❐ Long distances
❐ living nearby nature	
❐ Other: ______________________________	❐ Other: ______________________________

This part is about your experiences with teaching:

8. Do you agree with following sentences about your general attitude toward family medicine?

	strongly disagre e				strongly agree
	**1**	**2**	**3**	**4**	**5**
Doctors working in family medicine had a negative influence on my attitude toward general practice	☐	☐	☐	☐	☐
Doctors working in family medicine had a positive influence on my attitude toward general practice	☐	☐	☐	☐	☐
Doctors working in other specialist areas had a negative influence on my attitude toward general practice	☐	☐	☐	☐	☐
Doctors working in other specialist areas had a positive influence on my attitude toward general practice	☐	☐	☐	☐	☐

9. What did you generally find was the attitude toward family medicine of other medical specialists during your university education?

rather disparaging	☐ 1	☐ 2	☐ 3	☐ 4	☐ 5	rather appreciative

10. In retrospect, how would you assess the following courses in general practice?

	very bad				very good
	**1**	**2**	**3**	**4**	**5**
a) Course in general practice	☐	☐	☐	☐	☐
b) Block internship in general practice	☐	☐	☐	☐	☐
c) Lectures in the field of general practice<br>☐ did not attend any	☐	☐	☐	☐	☐
d) Further curricular courses in the field of general practice (Introduction to careers in medicine, Introduction to clinical medicine, Interdisciplinary areas)	☐	☐	☐	☐	☐

	very bad				very good
	1	2	3	4	5
e) Electives:<br>☐ did not attend any<br>1. ___________________________<br>2. ___________________________	 <br>☐<br>☐	 <br>☐<br>☐	 <br>☐<br>☐	 <br>☐<br>☐	 <br>☐<br>☐
f) Clinical traineeship in general practice<br>☐ Not completed<br>☐ Mandatory clinical traineeship<br>☐ Voluntary clinical traineeship	☐	☐	☐	☐	☐

11. Which courses in general changed your attitude toward family medicine in a positive or negative way?

**Positive**	**Negative**
**1.** ___________________________________	**1.** ___________________________________
**2.** ___________________________________	**2.** ___________________________________

12. Does a role model exist among the general practitioners you have met in your life?

☐ Yes ☐ No

13. Do you agree with following sentences?

	strongly disagree				strongly agree
	1	2	3	4	5
During my block internship, I had the opportunity to care for patients independently.	☐	☐	☐	☐	☐
If anything, I felt that excessive demands were made of me during my block internship.	☐	☐	☐	☐	☐
If anything, I felt that too few demands were made of me during my block internship.	☐	☐	☐	☐	☐
I was able to develop my communicative and social capabilities during my block internship in general practice.	☐	☐	☐	☐	☐
I felt like part of the practice team during my block internship in general practice.	☐	☐	☐	☐	☐

	strongly disagree				strongly agree
	1	2	3	4	5
During the block internship, my teaching physician found time to answer any questions and to teach me.	☐	☐	☐	☐	☐
During the block internship, my teaching physician seemed very happy in his profession.	☐	☐	☐	☐	☐
The courses in general practice were useful for my medical education.	☐	☐	☐	☐	☐
The courses in general practice provided me with sufficient insight into the subject to rule it out or consider it as a medical specialty.	☐	☐	☐	☐	☐

14. **In your general practice education, what topic areas do you think should be dealt with in more detail?** (Multiple choice possible)

- ❒ Economic aspects of setting up a practice
- ❒ Running and organizing a practice
- ❒ Conducting a conversation (e.g. bearing bad news, communicating with difficult patients)
- ❒ The path to setting up one's own practice
- ❒ Different working arrangements in general practice (group practices, employment, healthcare centers (Medizinisches Versorgungszentrum))
- ❒ The future of family medicine
- ❒ Internships in outpatient services to reduce uncertainty
- ❒ Frequent reasons for seeking advice concerning family medicine
- ❒ Additional titles (e.g.naturopathy)
- ❒ Structured specialist training in an organized network
- ❒ Working as a country doctor
- ❒ Other: ______________________________
- ❒ No information was missing in my general practice training
- ❒ There were too many courses in general practice

15. **In your opinion, in what form should additional educational content in general practice be taught?**

☐ Preferably in the form of voluntary courses ☐ Preferably in the form of mandatory courses

This part is about your image of family medicine:

16. How attractive or unattractive do you see following aspects of family medicine?

	Not attractiv e at all				Very attractiv e
	1	2	3	4	5
Value of family medicine in health care system	☐	☐	☐	☐	☐
Working conditions	☐	☐	☐	☐	☐
Compatibility of work and family	☐	☐	☐	☐	☐
Prevention	☐	☐	☐	☐	☐
Varied scope of diseases	☐	☐	☐	☐	☐
Focus on individual	☐	☐	☐	☐	☐
Lack of emergency situations	☐	☐	☐	☐	☐
Necessity of referral	☐	☐	☐	☐	☐
Focus on communication	☐	☐	☐	☐	☐
Less operations/ diagnostic	☐	☐	☐	☐	☐
Patients of all ages	☐	☐	☐	☐	☐
Many similar diseases	☐	☐	☐	☐	☐
Home visits	☐	☐	☐	☐	☐
Unlimited reachability	☐	☐	☐	☐	☐
On-call-duty	☐	☐	☐	☐	☐
Smaller Team	☐	☐	☐	☐	☐
Income	☐	☐	☐	☐	☐
Additional titles (e.g.naturopathy)	☐	☐	☐	☐	☐
Early decision between outpatient and inpatient patient care	☐	☐	☐	☐	☐
Prestige among colleagues	☐	☐	☐	☐	☐

	Not attractive at all				Very attractive
	1	2	3	4	5
Public prestige of family medicine	☐	☐	☐	☐	☐
Other: ___________________________________	☐	☐	☐	☐	☐

17. How important would be a change of following frame conditions in family medicine, to make it more attractive?

	Not important at all				Very important
	1	2	3	4	5
More possibilities of employment	☐	☐	☐	☐	☐
Training for taking over or founding a praxis	☐	☐	☐	☐	☐
Guarantee a professional representation	☐	☐	☐	☐	☐
Better coordination of medical specialist further education	☐	☐	☐	☐	☐
Guarantee a child care	☐	☐	☐	☐	☐
Support regarding finances	☐	☐	☐	☐	☐
Support regarding law and administration	☐	☐	☐	☐	☐
Support in learning competences of family medicine	☐	☐	☐	☐	☐
Optional courses to discuss questions concerning family medicine	☐	☐	☐	☐	☐
Other:	☐	☐	☐	☐	☐

Personal anonymized data:

18. Gender: ☐ female ☐ male

19. Age: ___________ years

20. Family status:

- ☐ married
- ☐ in partnership
- ☐ divorced
- ☐ single
- ☐ widowed

21. Do you have children?

☐ Yes ☐ No

22. Do you have a pre-education?

- ☐ Yes ____________________________
- ☐ No

23. How were you allowed to medical studies?

- ☐ Wait rate
- ☐ High school best quote
- ☐ Special admission strategies of universities
- ☐ Other:

____

24. a) Which is the size of municipality where you mainly grew up?

- ☐ Rural district (up to 5,000 inhabitants)
- ☐ Small town (>5,000 to 20,000 inhabitants)
- ☐ Medium-sized town (>20,000 to 100,000 inhabitants)
- ☐ Large town (>100,000 inhabitants)

b) Would you classify your personal background as predominantly rural?

☐ Yes ☐ No

25. a) Where were you born?

- ☐ In Germany; go to **26**
- ☐ In another country; go to **25 b)**

b) For how many years do you live in Germany?

____________ years

26. Where did your parents grow up?

Your <u>father:</u>

- ☐ Mainly in Germany
- ☐ Mainly in another country

Your mother:

- ☐ Mainly in Germany
- ☐ Mainly in another country

Thank you!

☺
